# Deep learning based on dynamic susceptibility contrast MR imaging for prediction of local progression in adult-type diffuse glioma (grade 4)

**DOI:** 10.1038/s41598-023-41171-9

**Published:** 2023-08-24

**Authors:** Donggeon Heo, Jisoo Lee, Roh-Eul Yoo, Seung Hong Choi, Tae Min Kim, Chul-Kee Park, Sung-Hye Park, Jae-Kyung Won, Joo Ho Lee, Soon Tae Lee, Kyu Sung Choi, Ji Ye Lee, Inpyeong Hwang, Koung Mi Kang, Tae Jin Yun

**Affiliations:** 1 Department of Radiology, Seoul National University College of Medicine, Seoul, Republic of Korea; 2 Department of Radiology, Seoul National University Hospital, 101, Daehangno, Jongno-Gu, Seoul, 03080 Republic of Korea; 3 Center for Nanoparticle Research, Institute for Basic Science (IBS), Seoul, Republic of Korea; 4 School of Chemical and Biological Engineering, Seoul National University, 1, Gwanak-Ro, Gwanak-Gu, Seoul, 302-909 Republic of Korea; 5 Department of Internal Medicine, Cancer Research Institute, Seoul National University College of Medicine, Seoul, Korea; 6 Department of Neurosurgery, Biomedical Research Institute, Seoul National University College of Medicine, Seoul, Korea; 7 Department of Pathology, Seoul National University College of Medicine, Seoul, Korea; 8 Department of Radiation Oncology, Cancer Research Institute, Seoul National University College of Medicine, Seoul, Korea; 9 Department of Neurology, Seoul National University College of Medicine, Seoul, Korea

**Keywords:** Oncology, Nervous system

## Abstract

Adult-type diffuse glioma (grade 4) has infiltrating nature, and therefore local progression is likely to occur within surrounding non-enhancing T2 hyperintense areas even after gross total resection of contrast-enhancing lesions. Cerebral blood volume (CBV) obtained from dynamic susceptibility contrast perfusion-weighted imaging (DSC-PWI) is a parameter that is well-known to be a surrogate marker of both histologic and angiographic vascularity in tumors. We built two nnU-Net deep learning models for prediction of early local progression in adult-type diffuse glioma (grade 4), one using conventional MRI alone and one using multiparametric MRI, including conventional MRI and DSC-PWI. Local progression areas were annotated in a non-enhancing T2 hyperintense lesion on preoperative T2 FLAIR images, using the follow-up contrast-enhanced (CE) T1-weighted (T1W) images as the reference standard. The sensitivity was doubled with the addition of nCBV (80% vs. 40%, *P* = 0.02) while the specificity was decreased nonsignificantly (29% vs. 48%, *P* = 0.39), suggesting that fewer cases of early local progression would be missed with the addition of nCBV. While the diagnostic performance of CBV model is still poor and needs improving, the multiparametric deep learning model, which presumably learned from the subtle difference in vascularity between early local progression and non-progression voxels within perilesional T2 hyperintensity, may facilitate risk-adapted radiotherapy planning in adult-type diffuse glioma (grade 4) patients.

## Introduction

Despite multimodal treatment including maximal surgical resection, radiation, and chemotherapy, adult-type diffuse glioma (grade 4) remains the most aggressive primary brain tumor, with an average survival of 12–15 months due to the high rate of progression^1–3^. One of the most lethal characteristics of adult-type diffuse glioma (grade 4) is its infiltrating nature, which leads to the consensus that borders of contrast-enhancing tumors are not the true margins of adult-type diffuse glioma (grade 4)^4^. Consequently, local progression is likely to occur within surrounding non-enhancing T2 hyperintense areas after gross total resection of contrast-enhancing lesions. Thus, it has been emphasized that local control of adult-type diffuse glioma (grade 4), along with systemic treatments, is crucial to improve the survival of adult-type diffuse glioma (grade 4) patients^5^.

There have been variable approaches to predict local progression of adult-type diffuse glioma (grade 4) using advanced MRI. Early studies have shown that diffusion tensor imaging (DTI), diffusion-weighted imaging (DWI), and positron emission tomography (PET) may help predict recurrent adult-type diffuse glioma (grade 4)^6–8^.

Cerebral blood volume (CBV) obtained from dynamic susceptibility contrast perfusion-weighted imaging (DSC-PWI) is a parameter that is well-known to be a surrogate marker of both histologic and angiographic vascularity in tumors^9,10^. There have been several attempts to evaluate the potential of DSC-PWI in predicting local progression and survival after chemoradiation in adult-type diffuse glioma (grade 4) patients^11–14^. Specifically, a radiomics study with DSC-PWI and DTI has demonstrated that radiomics analysis of fractional anisotropy and normalized CBV (nCBV) in the peritumoral non-enhancing region has the potential to predict local progression and survival in adult-type diffuse glioma (grade 4) patients^12^. Furthermore, a recent study showed that deep learning models that utilize high-dimensional radiomics profiles based on nCBV maps can help predict local progression and distant progression in adult-type diffuse glioma (grade 4)^14^.

To our knowledge, however, no research has focused on predicting early local progression by using a multiparametric deep learning model based on DSC-PWI features. Early local progression within one year was chosen as the primary outcome because the median time to progression was reported to be 5.3 months in patients with newly diagnosed glioblastoma, implying the majority of patients had progression prior to six months^15^. The purpose of our study was to compare the diagnostic performances of a deep learning model based on multiparametric MRI, including DSC-PWI, and that based on conventional MRI alone for predicting early local progression of adult-type diffuse glioma (grade 4).

## Results

### Patient characteristics

The clinical features of the early local progression and non-progression groups are presented in Table 1. O6-methylguanine DNA methyltransferase (MGMT) promoter methylation was more common in the non-progression group (70% [87 of 124]) than in the early local progression group (30% [26 of 88]) (*P* < 0.001). The patients in the early local progression group had a higher incidence of IDH wildtype than those in the non-progression group (96% [84 of 88] vs. 87% [108 of 124], respectively; *P* = 0.03). There were no statistically significant differences in age or sex between the two groups (*P* > 0.05). The clinical features of the training and test sets are shown in Supplementary Table 1.

**Table 1:** Clinical characteristics of the early local progression and non-progression groups.

Characteristics	Total (n = 212)	Early local progression (n = 88)	Non-progression (n = 124)	*P* value
Mean age (years)^a^	57.8 ± 13.4	59.9 ± 12.7	56.4 ± 13.8	0.06^b^
Sex				
Male	112 (53)	51 (58)	61 (49)	0.13^c^
Female	100 (47)	37 (42)	63 (51)	
Methylated MGMT promoter				
Positive	113 (53)	26 (30)	87 (70)	< 0.001^c^
Negative	99 (47)	62 (70)	37 (30)	
IDH1/2 mutation				
Positive	17 (8)	3 (3)	14 (11)	0.03^c^
Negative	192 (91)	84 (96)	108 (87)	
Not available	3 (1)	1 (1)	2 (2)	

*MGMT*, O^6^-methylguanine-DNA methyltransferase, *IDH* isocitrate dehydrogenase.

Unless otherwise indicated, data represent the number of patients (percentages).

^a^Data are means ± SD.

^b^Calculated with the independent samples t-test.

^c^Calculated with Fisher’s exact test.

### Computational time

Diagnostic performance of the conventional MRI model converged after approximately 585 epochs of 38 h by Adam optimizer with learning rate weight decay^16^. The multiparametric MRI model performance converged after approximately 309 epochs of 32 h (Supplementary Fig. 1).

### Diagnostic performance of deep learning models

**Figure 1:**
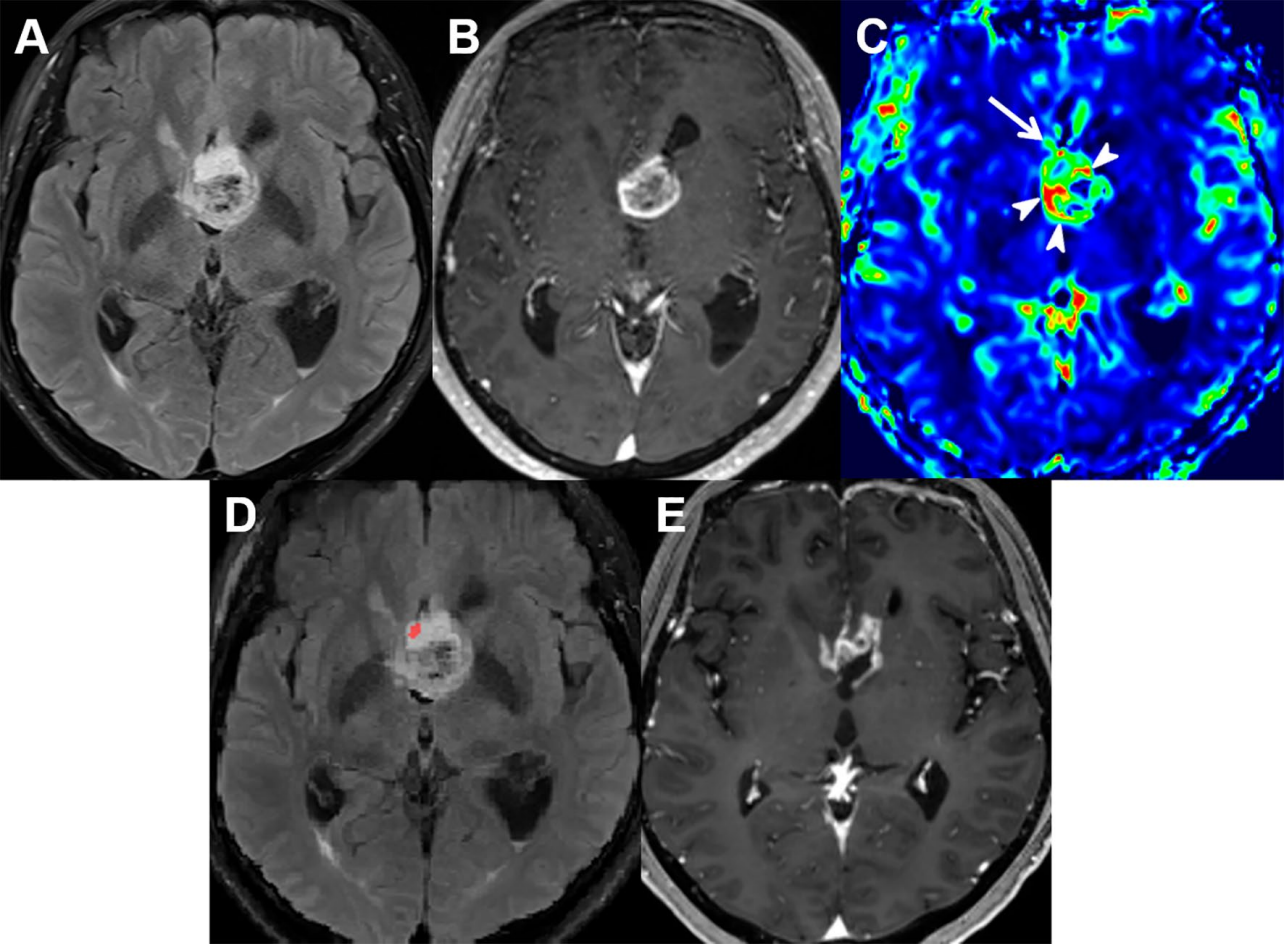
A 58-year-old woman with glioblastoma in the training set. (**A,B**) On axial preoperative T2 FLAIR and CE T1W images, a heterogeneously enhancing mass was found at the septum pellucidum and corpus callosum along with a small surrounding area of non-enhancing T2 hyperintensity. (**C**) nCBV is mildly increased at the anterior aspect (arrow) of the enhancing tumor, which shows an overt increase in nCBV (arrowheads). (**D**) The multiparametric MRI model predicted early local progression to occur at the red area anterior to the enhancing tumor. (**E**) Follow-up MR images at 7 months confirmed local progression at the corpus callosum. *FLAIR* fluid-attenuated inversion recovery, *CE T1W* contrast-enhanced T1-weighted.

**Figure 2:**
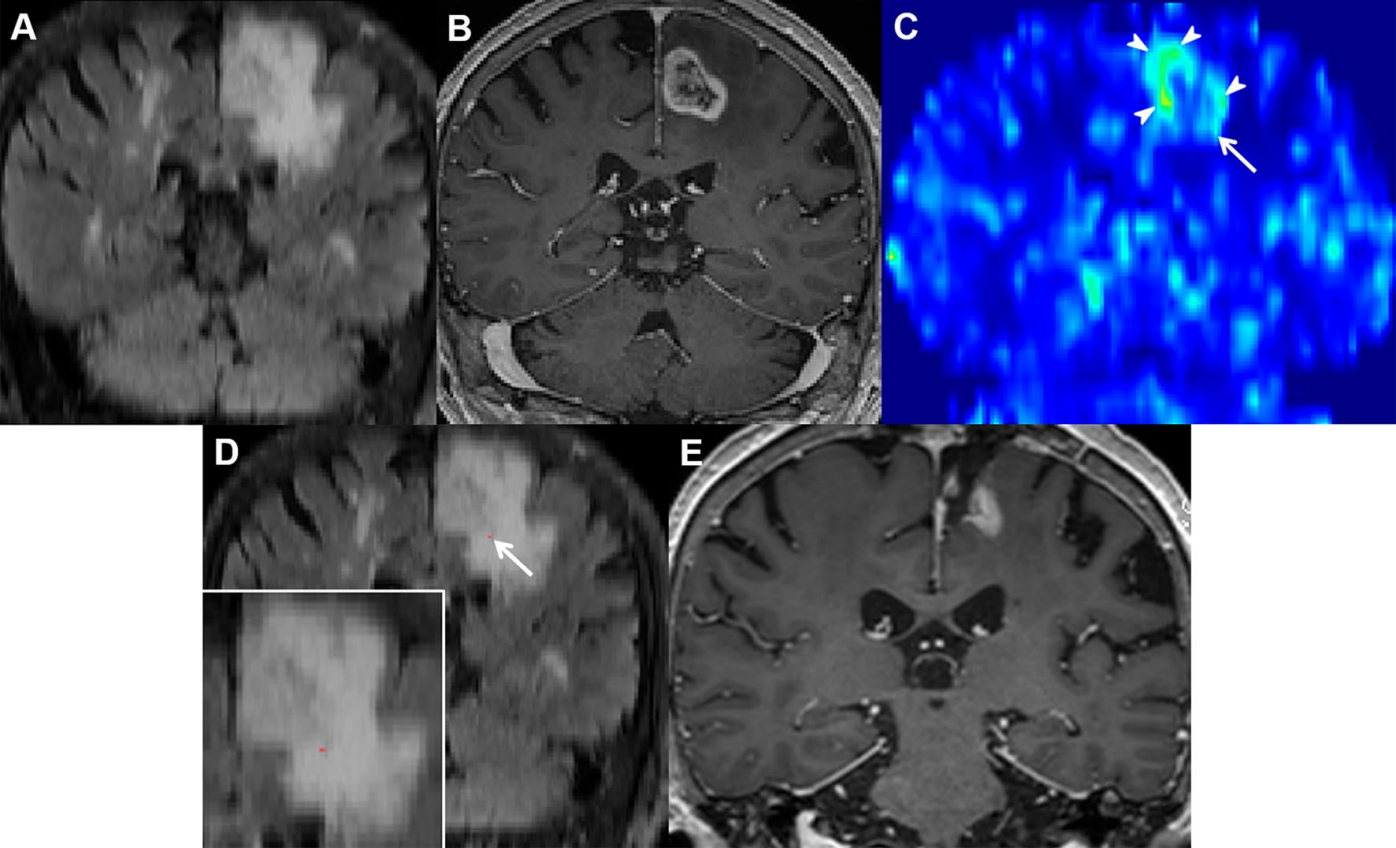
A 71-year-old woman with glioblastoma in the test set. (**A,B**) On coronal preoperative T2 FLAIR and CE T1W images, a heterogeneously enhancing mass was noted in the left parietal lobe along with extensive perilesional T2 hyperintensity. (**C**) The nCBV map depicts a focal mild increase at the inferior aspect of the enhancing tumor (arrow) along with a definite increase at the enhancing portion of the tumor (arrowheads). (**D**) The multiparametric MRI model predicted early local progression to occur at the red area inferior to the enhancing tumor (magnified view at the lower right corner). (**E**) Follow-up MR images at 7 months demonstrated a measurable enhancing lesion at the inferior aspect of the surgical cavity. *FLAIR* fluid-attenuated inversion recovery, *CE T1W* contrast-enhanced T1-weighted.

In the training set, the multiparametric MRI model had a higher sensitivity for predicting early local progression than the conventional MRI model (69% [47 of 68] vs. 62% [42 of 68], respectively; *P* = 0.49), although the statistical significance was not reached. The specificity in predicting early local progression did not significantly differ between the two deep learning models (26% [27 of 103] for the multiparametric MRI model vs. 32% [33 of 103] for the conventional MRI model; *P* = 0.42). In the test set, the multiparametric MRI model had a significantly higher sensitivity (80% [16 of 20]) for the prediction of early local progression than the conventional MRI model (40% [8 of 20]) (*P* = 0.02). The specificity was nonsignificantly decreased in the multiparametric MRI model, as compared with the conventional MRI model (29% [6 of 21] vs. 48% [10 of 21], respectively; *P* = 0.39) (Table 2). (Receiver operating characteristic curves showing sensitivities and specificities at various probability cutoff values in the test set are provided in Supplementary Fig. 2.) The accuracies did not significantly differ between the conventional and multiparametric MRI models in both training (44% [75 of 171] vs. 43% [74 of 171], respectively; *P* = 1.00) and test (44% [18 of 41] vs. 54% [22 of 41], respectively; *P* = 0.52) sets. Figures 1 and 2 show representative cases of early local progression prediction in the training and test sets.

**Table 2:** Diagnostic performance of deep learning models.

	Conventional model	Multiparametric model	*P* value
Sensitivity	40% (8 of 20)	80% (16 of 20)	0.02
Specificity	48% (10 of 21)	29% (6 of 21)	0.39

Numbers in parentheses are raw data.

## Discussion

In this study, we established and validated two deep learning models for the prediction of early local progression in adult-type diffuse glioma (grade 4), one based on conventional MRI alone and the other based on the combination of conventional MRI and the nCBV map from DSC-PWI. We found that the deep learning model based on multiparametric MRI, including DSC-PWI, had a significantly higher sensitivity than the conventional MRI model while having a similar specificity.

DSC-PWI can provide nCBV, one of the most reliable markers to evaluate microvascular attenuation and tumor vasculature, which cannot be determined by conventional MRI. Given that nCBV is increased in high-grade gliomas due to active neoangiogenesis^9,10,17,18^, vascularity information could be used to differentiate infiltrated tumor cells with a high probability of future local progression from perilesional edema. This may explain why the addition of the nCBV map led to the marked improvement in the sensitivity of the multiparametric MRI model for predicting early local progression, as compared to the conventional MRI model. Our results are consistent with those in the literature, in which a predictive support vector machines (SVM) model for tumor infiltration and future recurrence based on multiparametric MRI, including DSC-PWI, had higher diagnostic performance than that based on conventional MRI alone^19^. Kim et al. also reported that only radiomics features extracted from nCBV of peri-tumoral non-enhancing regions, such as covered image intensity range and mean contrast/inertia, (but not those from conventional MRI) were finally included in a diagnostic model to predict 6-month progression, in keeping with the results of our study^12^. In addition, Lundemann et al. also reported that recurring voxels within non-enhancing lesions had higher blood volume calculated from DCE-MRI than non-recurring voxels, highlighting the clinical value of the perfusion characteristics^7^.

As adult-type diffuse glioma (grade 4) are highly aggressive, angiogenic tumors, their growth displays a considerable amount of neovascularization, which is reflected by increased nCBV in contrast-enhancing portions with high cellularity^9,10,17,18^. However, the change in nCBV in non-enhancing T2 hyperintense lesions, induced by infiltrated tumor cells intermingled with peritumoral edema, is often too subtle to be recognized by visual assessment of nCBV with the naked eye^20^. Previous attempts have been made to overcome this limitation by using SVM algorithms to evaluate the time series of DSC-PWI data that convey different aspects of the dynamics of blood perfusion (e.g., baseline signal, depth of signal decrease, slope of signal decrease and recovery)^19–21^. In particular, using multiparametric MRI including DTI and perfusion temporal dynamics data from DSC-PWI, SVM generated spatial maps representing the likelihood of tumor infiltration, which correlated well with regions of recurrence in postresection follow-up studies^19^. The radiomic signature of the recurrent tumor region reflected higher vascularity and cellularity, as compared to the nonrecurrent region^19^. Deep learning networks, consisting of many hidden layers and multiple neurons per layer, use a large amount of ground truth designated data to find unique features and their combinations for integrated feature extraction and to construct classification models^22^. One of the main differences between deep learning and machine learning techniques is that deep learning can create new features by itself, whereas machine learning requires the features to be accurately and precisely recognized by users^22^. In this study, we were able to build an nnU-net-based prediction model that presumably learned from the subtle difference in vascularity between the early local progression and non-progression voxels within perilesional T2 hyperintensity. Our study differs from the previous studies in that we used MR images that are more readily available in routine practice for tumor evaluation (i.e., CE T1W images, FLAIR images, and nCBV maps) and a deep learning algorithm instead of a machine learning algorithm to build a predictive model with a high sensitivity for early local progression.

With regard to the clinical implications, the deep learning model predictive of early local progression may facilitate risk-adapted radiotherapy planning, in which patients predicted to have early local progression by the model are treated with a higher radiation dose for better local control. In terms of the diagnostic performance, the sensitivity was doubled with the addition of nCBV, while the specificity was decreased nonsignificantly, implying that fewer cases of early local progression would be missed with the multiparametric MRI model, although more cases of non-progression would receive unnecessarily high radiation doses. Unnecessarily high radiation doses can increase the rate of radiation-induced complications; nonetheless, we speculate that the clinical consequence of missing patients with a high risk of early local progression who may have a survival gain from increased radiation doses would be greater in routine practice. Moreover, given the high rate of early local progression in adult-type diffuse glioma (grade 4), we expect the deep learning algorithm with a higher sensitivity (albeit lower specificity) would result in a higher clinical benefit. Of note, the sensitivity of the model may be impressive in that the model was not provided with any surgical information during training and thus it essentially predicted not only the likelihood of early local progression at a specific location but also the chance of those being included in a supratotal resection. However, although there were some cases that were correctly predicted to have early local progression with the addition of CBV information, the accuracy of the multiparametric MRI model was still low, which may reflect the difficulty of the prediction task.

With regard to the specificities of the models, susceptibility variation can influence the specificities of both inversion-based T1 sequences such as MPRAGE and DSC-PWI. Specifically, DSC-PWI exploits the regional susceptibility-induced signal loss caused by paramagnetic gadolinium-based contrast agents, while contrast volume and tissue morphology can add susceptibility-related T2 * signal loss in T1 enhancement in inversion-based T1 sequences. In addition to the tissue morphology and contrast volume, non-biological heterogeneity due to hardware (magnetic field strength, MRI vendor) and software or MRI protocol variation are also sources of susceptibility variations. These variations may be a potential source of the relatively low specificity of the model with DSC-PWI as compared with the conventional MR model, given that the variation is less in conventional MR sequences such as T2 FLAIR imaging that refocus susceptibilities. Therefore, such variations hinder the accurate comparison of signal intensities from inversion-based T1 sequences such as MPRAGE and DSC-PWI between studies, and ideally, normalization using contralateral normal tissue for each patient during any particular session and normalization using a standardized brain gel phantom are required for better comparison.

As for MR imaging techniques, we have used DSC-PWI − a more commonly used PWI technique − in this study, instead of dynamic contrast-enhanced (DCE) or arterial spin labeling (ASL) MR imaging. Baseline DCE MR parameters of non-enhancing T2 high signal intensity lesions may have prognostic values and predict the recurrence in grade 4 adult-type diffuse glioma patients undergoing the standard treatment^23–26^. Moreover, ASL perfusion patterns at non-enhancing portions of tumors have been reported to be significant predictors for progression-free and overall survival^27^. Given the prognostic values of DCE and ASL MR imaging, the possibility exists that deep learning models which learn from DCE or ASL MR imaging may have better diagnostic performance than that based on DSC-PWI. Future studies based on various PWI data are warranted for the comparison. In addition, we used an ultrafast GRE MR sequence with magnetization-preparation to shorten MR scanning time. However, despite the relatively low contrast between gray and white matter on 3D T1-weighted TSE MR sequences than on ultrafast GRE MR sequences with magnetization-preparation, post-contrast 3D T1-weighted TSE sequences are known to be more sensitive for the detection of contrast-enhancing lesions due to following reasons: (a) improved image contrast between contrast-enhancing lesions and surrounding brain parenchyma; (b) higher signal-to-noise-ratio; (c) intrinsic black blood effect; and (d) less artifacts from static field inhomogeneity^28^. In this study, regions-of-interest denoting the area of future local recurrence were drawn within non-enhancing T2 hyperintense lesion on preoperative T2 FLAIR images. The use of 3D T1-weighted TSE MR sequences instead of ultrafast GRE MR sequences may facilitate the distinction between contrast-enhancing and non-enhancing areas within tumors.

Apart from intrinsic limitations of a retrospective study, our study has several limitations. First, instead of external validation, a temporal validation strategy was used to provide some information on the reproducibility and generalizability of our models, and thus a future prospective multicenter study is warranted to provide stronger evidence for the generalizability of the models. Second, local progression was labelled on preoperative T2 FLAIR images using follow-up MR images as the reference standards, but the change in geometry due to anatomical distortion following surgery could have resulted in some mismatch between annotated labels and true local progression area on follow-up MR images. We minimized these voxels by cross-checking the labels with expert radiologists with more than 12 years of experience. Third, the time information of local progression (e.g., time to progression) was not taken into account when analyzing local progression and could be incorporated into future models to refine their prognostic information. Fourth, although the prognosis varies among ‘grade 4 adult-type diffuse gliomas’ according to the IDH mutation status^29–34^, we grouped them together to develop the deep learning models in this study. Grade 4 adult-type diffuse gliomas are characterized by their infiltrative growth, and therefore, have infiltrative tumor cells within the non-enhancing T2 hyperintensity regardless of the IDH mutation status^35^. Moreover, the standard treatment strategies are also the same for both ‘glioblastoma, IDH-wildtype, grade 4’ and ‘astrocytoma, IDH-mutant, grade 4’ at present^36^. A future study based on a larger dataset is needed to develop two separate models according to the IDH mutation status. Fifth, given that the development of reliable deep learning algorithms often require a large dataset, we focused on the added value of DSC-PWI, a more commonly used advanced MR technique. A future study is needed to test whether adding other advanced MR techniques such as amide chemical exchange saturation transfer (CEST) imaging or dynamic contrast-enhanced MR imaging could further improve the diagnostic performance of our multiparametric model. Sixth, although we have normalized our pixel-based CBV maps using the unaffected white matter, normalization using a standardized brain gel phantom periodically to correct patient data week to week would be more desirable for better comparison of signal intensities from inversion-based T1 sequences such as MPRAGE and DSC-PWI between studies. Seventh, given the variation in sensitivities and specificities according to the probability cutoff values, the possibility exists that the difference in sensitivities and specificities observed may be due to chance in this small study population. Nonetheless, an AUC value of the multiparametric MRI model tended to be higher than that of the conventional MRI model, suggesting the potential added value of nCBV. A further study based on a larger sample size is needed to validate the added value of nCBV.

In conclusion, we developed a deep learning model based on multiparametric MRI, including DSC-PWI, which was superior to the model based on conventional imaging alone in terms of sensitivity for the prediction of early local progression in adult-type diffuse glioma (grade 4) patients. While the accuracy of CBV model is still poor and needs improving, this study illustrates how deep learning-based models using CBV might facilitate risk-adapted radiotherapy planning for better local control in adult-type diffuse glioma (grade 4) patients.

## Materials and methods

The Institutional Review Board of Seoul National University Hospital approved this retrospective study and waived the requirement for informed consent. The study protocol was performed in accordance with the Declaration of Helsinki.

### Patients

Six hundred and eight consecutive patients diagnosed with adult-type diffuse glioma (grade 4) from May 2010 to February 2022 at Seoul National University Hospital were enrolled in this study.

The inclusion criteria were as follows: the patient (a) had a histopathologic diagnosis of glioblastoma, IDH-wildtype, grade 4 or astrocytoma, IDH-mutant, grade 4 based on the 2021 World Health Organization (WHO) criteria; Ref.^37^ (b) had undergone the standard treatment (i.e., maximal surgical resection followed by radiation therapy with concurrent temozolomide (TMZ) and adjuvant TMZ); (c) had preoperative and follow-up 3 T MRI including contrast-enhanced (CE) T1-weighted (T1W) imaging, DSC-PWI, and fluid-attenuated inversion recovery (FLAIR) imaging; and (d) had early local progression within 1 year or had been followed up for more than 1 year after surgery.

The exclusion criteria were as follows: (a) subtotal resection or biopsy (n = 179); (b) inadequate image quality or incomplete imaging data for analysis (n = 122); (c) follow-up loss (n = 89); and (d) age younger than 18 years (n = 6). Under these inclusion and exclusion criteria, a total of 212 patients with gross total resection of contrast-enhancing lesions were finally included in our study. Imaging-based gross total resection was defined as no residual measurable enhancing lesion on the immediate postoperative MRI taken within 24 to 48 h after surgery (according to RANO guideline^38^), apart from the linear enhancement along the wall of surgical cavity. In addition, gross total resection was clinically considered to be achieved when there was no residual 5-aminolevulinic acid (5-ALA)-induced tumor fluorescence in the resection margin^39^.

The MRI data were split into two datasets based on the time of initial diagnosis of grade 4 astrocytoma: a training set (n = 171) with the diagnosis made between 2010 and 2019 and a test set (n = 41) with the diagnosis made between 2020 and 2022. The patients were classified into ‘early local progression’ and ‘non-progression’ groups with respect to the presence of local progression within 1 year after the surgery. We defined local progression as the development of a new measurable contrast-enhancing lesion within the T2 hyperintense area surrounding a surgical cavity margin in this study^38^.

**Figure 3:**
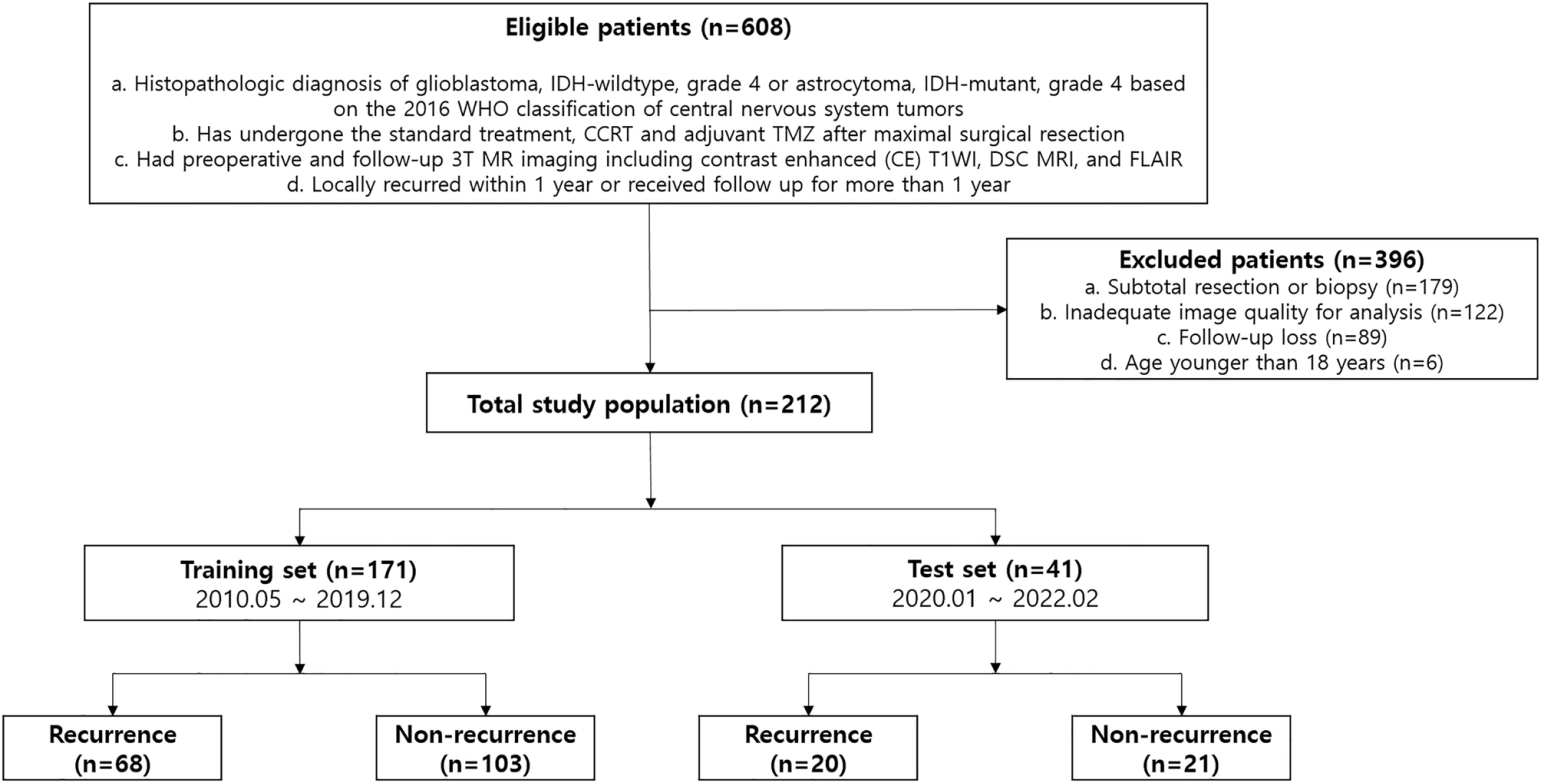
Flowchart for patient selection and classification.

As a result, our final study population was categorized into the early local progression group (total [n = 88]; training set [n = 68], test set [n = 20]) and the non-progression group (total [n = 124]; training set [n = 103], test set [n = 21]) (Fig. 3). Clinical variables, such as age, sex, O6-methylguanine-DNA methyltransferase (MGMT) promoter methylation status, and isocitrate dehydrogenase (IDH) mutation, were recorded.

### MRI protocol

All MRI was performed at a 3.0 T imaging unit with a 32-channel head coil (Magnetom Verio [n = 115], Siemens Healthineers; Magnetom Skyra [n = 65], Siemens Healthineers; Magnetom Trio [n = 2], Siemens Healthineers; Ingenia CX 3.0 T [n = 26], Philips Healthcare; Discovery MR 750w [n = 4], GE Healthcare). The MRI protocol included the 3D T1W magnetization-prepared rapid acquisition gradient echo sequence (MPRAGE) before and after injection of gadobutrol (Gadovist; Bayer, Berlin, Germany; at a dose of 0.1 mmol/kg of body weight), T2 FLAIR, and DSC-PWI.

DSC-PWI was performed using the following parameters: repetition time (TR) = 1500 − 1600 ms; echo time (TE) = 29.3 − 40 ms; flip angle = 35 − 90°; matrix = 100 × 100 or 128 × 128; FOV = 240 × 240 mm^2^; section thickness = 4 − 5 mm; and number of excitations = 1. Postprocessing of DSC-PWI was performed according to a previously published method^14^. To minimize the variance due to the difference in MRI scanners and protocols, pixel-based CBV maps were normalized by dividing every CBV value in a specific section by that in the unaffected white matter. Specific MR scan parameters for all MR sequences are provided in Supplementary Table 2.

### Deep learning algorithm development

#### Local progression labeling

**Figure 4:**
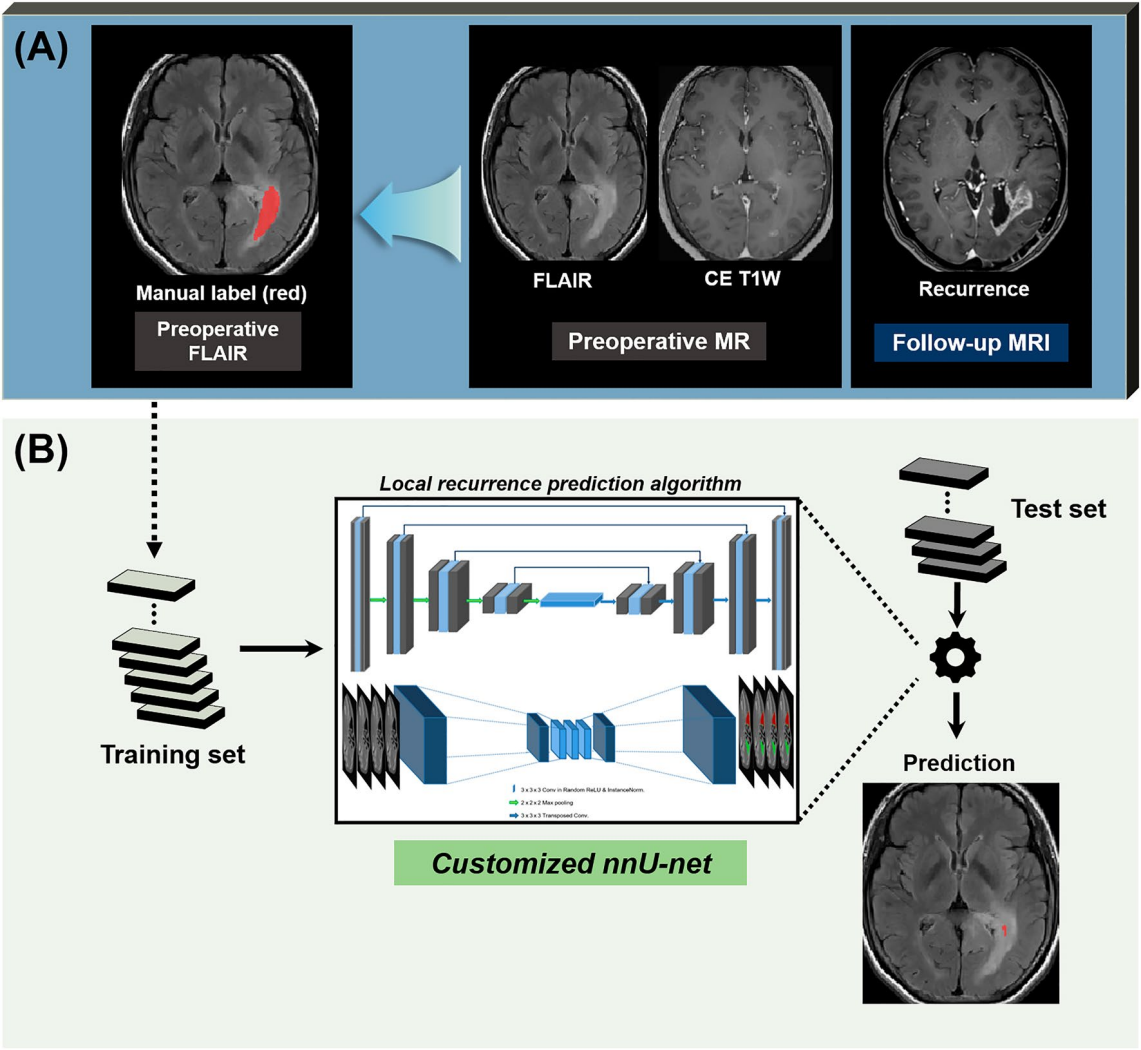
Development of a deep learning algorithm based on conventional MRI. (**A**) Using the follow-up CE T1W images as the reference standards, manual labels for local progression were drawn in each section of a non-enhancing T2 hyperintense lesion on preoperative T2 FLAIR images. Preoperative T2 FLAIR images and manual labels were coregistered to the resampled CE T1W images (not shown). (**B**) An nnU-Net based deep learning model was trained on conventional MRI alone, including FLAIR and CE T1W images. *FLAIR*, fluid-attenuated inversion recovery, *CE T1W* contrast-enhanced T1-weighted.

**Figure 5:**
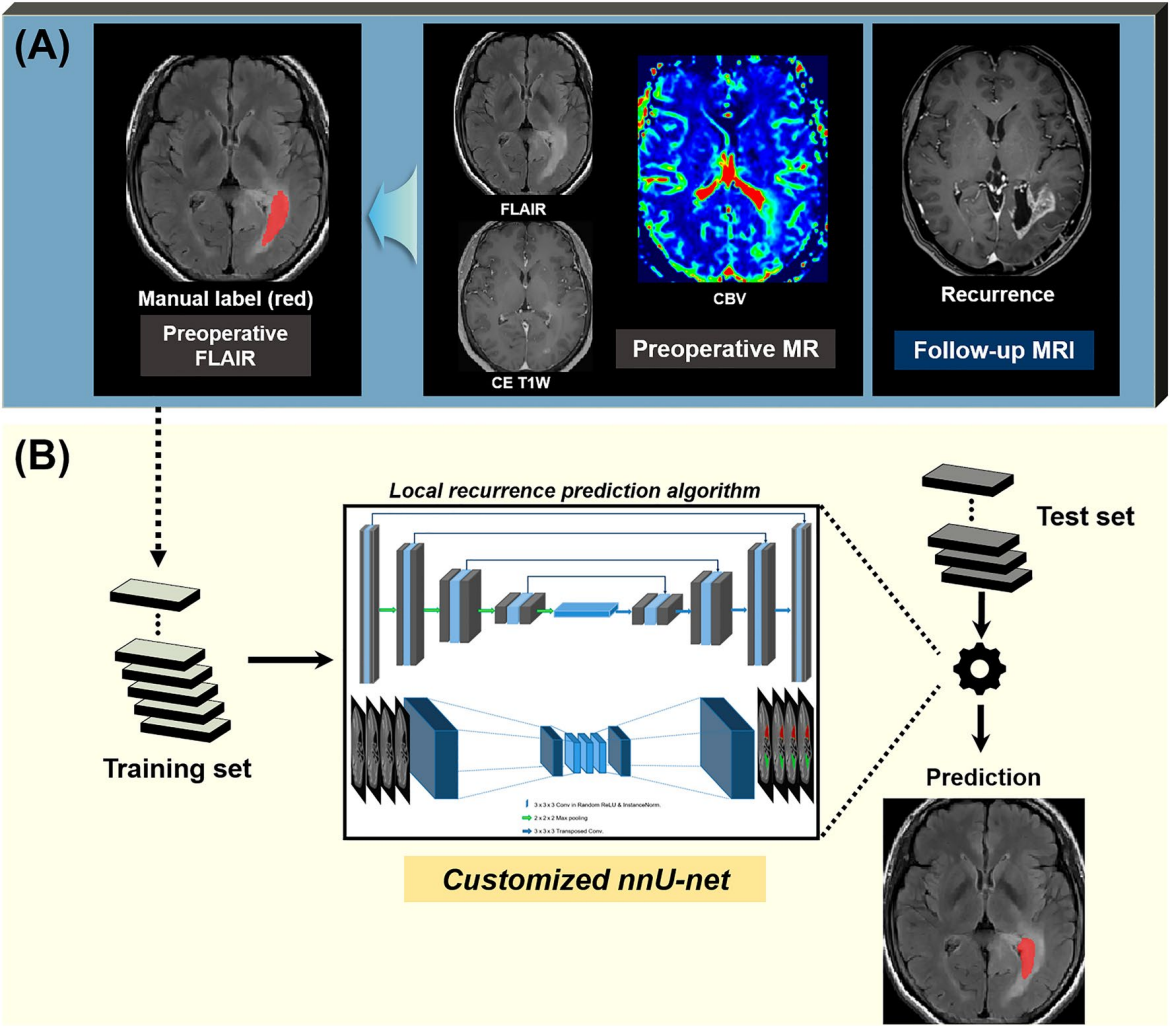
Development of a deep learning algorithm based on multiparametric MRI. (**A**) Using the follow-up CE T1W images as the reference standards, manual labels for local progression were drawn in each section of a non-enhancing T2 hyperintense lesion on preoperative T2 FLAIR images. Preoperative T2 FLAIR images, nCBV map, and mask images were coregistered to the resampled CE T1W images (not shown). (**B**) An nnU-Net based deep learning model was trained on multiparametric MRI, including CE T1W images, FLAIR images, and nCBV maps. *FLAIR* fluid-attenuated inversion recovery, *CE T1W* contrast-enhanced T1-weighted, *nCBV* normalized cerebral blood volume.

Labeling of the location of local progression in the training and test datasets was manually performed by investigators supervised by two expert radiologists (R.E.Y. and S.H.C. with 12 and 19 years of neuro-oncology imaging experience, respectively) using ITK-SNAP software (version 3.8.0, http://www.itksnap.org/pmwiki/pmwiki.php)^40^. Prior to the labeling, each set of follow-up MR images with clear radiological features of local progression was opened at the picture archiving and communication system (PACS) workstation, and contrast-enhancing lesions around surgical margins on CE T1W images were defined as the reference standards for the sites of local progression. Based on the reference standards, regions-of-interest (ROIs) were carefully drawn in each section of a non-enhancing T2 hyperintense lesion on preoperative T2 FLAIR images by the consensus of two radiologists, to define a specific subset of voxels within the non-enhancing T2 hyperintense lesion where tumor progression would occur (Figs. 4A and 5A).

#### Image preprocessing

First, CE T1W images were resampled to 1 mm iso-voxels with linear interpolation. Subsequently, other images, including FLAIR, manual labels drawn on FLAIR images by the experienced radiologists, and nCBV maps, were co-registered to the corresponding resampled CE T1W images, using BRAINSFit of 3D Slicer. Two expert radiologists (R.E.Y. and S.H.C. with 12 and 19 years of neuro-oncology imaging experience, respectively) manually checked and confirmed all co-registration results.

#### Deep learning model development

An nnU-Net deep learning algorithm was used to develop early local progression prediction models (Figs. 4B and 5B) on a workstation with NVIDIA GeForce RTX 3090 GPU. nnU-Net has the same basic neural network configuration as U-Net but concentrates its effort on pre/post data processing and hyperparameter setting for more practical use and better performance. A previous study has demonstrated that nnU-Net outperformed most existing approaches in a range of diverse tasks on 23 public datasets used in international biomedical segmentation competitions^41^. In particular, nnU-Net has the following advantages: (a) it automatically configures itself for any new task, including preprocessing, network architecture, training, and post-processing; (b) it can handle a wide variety of biomedical imaging datasets; (c) it does not require any user intervention; and (d) it is computationally feasible^41^.

In our model training, two separate models (conventional MRI model and multiparametric MRI model) were developed using different dataset inputs: CE T1W images and FLAIR images for the conventional MRI model and CE T1W images, FLAIR images, and nCBV maps for the multiparametric MRI model. Subsequently, hyperparameters related to model training were automatically determined by nnU-Net on the basis of the core characteristics of the dataset, such as class ratio image size and voxel spacing information. Full resolution 3D models were used for training rather than 2D models or cascade approaches because 2D models have been known to predict outcomes based on limited information as compared to 3D models. Working deep learning models were more easily obtained because nnU-Net covered from data augmentation to patch generation and patch stitching to obtain final prediction results. The computing time required to predict the presence of local progression (including data preprocessing time) for a single patient was within 2 − 3 min. (Details are provided in ‘Supplementary Materials’ and ‘Supplementary Table 3’).

### Statistical analysis

Statistical software (MedCalc, version 11.1.1.0, Mariakerke, Belgium) was used to perform all statistical analyses. The Kolmogorov–Smirnov test was used to check normality for each parameter. Differences in clinical characteristics between the early local progression and non-progression groups were analyzed using the Fisher’s exact test for categorical variables and the independent samples t-test for non-categorical variables. The sensitivity and specificity were compared between the conventional MRI model and the multiparametric MRI model, using the McNemar test. Voxels with the probability of ‘0.5’ were shown as the model output. To assess the sensitivity and specificity at the patient level, cases with at least one voxel were considered to have ‘local progression’ as the predicted result. *P* values less than 0.05 were considered to be statistically significant in all tests.

### Supplementary Information

Supplementary Information.

## Data Availability

The datasets generated during and/or analysed during the current study are available from the corresponding author on reasonable request.
