# Supplementary material for: Deep learning based on dynamic susceptibility contrast MR imaging for prediction of local progression in adult-type diffuse glioma (grade 4)
Source: Sci Rep. 2023 Aug 24;13:13864. doi: 10.1038/s41598-023-41171-9 (PMC10449894; doi:10.1038/s41598-023-41171-9)

Supplementary Materials

Deep Learning Model Development

The architecture of nnU-Net is based on the U-Net architecture, which includes an encoder and a decoder connected by a series of skip connections. The encoder comprises convolutional and pooling layers that progressively downsample the input image, while the decoder pathway uses upconvolutional and concatenation operations to gradually upsample the feature maps and recover the original image resolution. The skip connections connect corresponding layers of the encoder and decoder, enabling the decoder to access information from earlier stages of the network. Our network had six convolutional layers and five pooling layers, all with a kernel size of 3 × 3 × 3 for the convolutional layers and 2 × 2 × 1 for the first pooling layer followed by five 2 × 2 × 2.

The network uses a combination of two loss functions, namely the dice loss and the cross-entropy loss (Supplementary Table 3). The initial network weights are initialized using the Kaiming He method, and the initial learning rate is set to 0.0003, which decays over time using the Adam optimizer [1]. nnU-Net uses various data augmentation techniques to increase the size and diversity of the training set. The augmentation techniques included rotation, gamma correction, scaling, elastic deformation, and mirroring. The input images were divided into patches of size 128 × 128 × 112, which were fed into the network during training. The batch size of 2 was relatively small as compared with other approaches, and it was chosen based on the available GPU memory. The patch and batch size were determined based on the size and resolution of the input images. The model was trained using Python version 3.7 and PyTorch version 1.2.

Supplementary Tables

Supplementary Table 1. Clinical characteristics of the training and test sets.

Characteristics	Total (n = 212)	Training set (n = 171)	Test set (n = 41)	*P* value
**Mean age (years)**[a]	57.8 ± 13.4	57.5 ± 13.7	59.1 ± 11.9	0.19[b]
**Sex**				1.00[c]
Male	112 (53)	89 (52)	23 (56)	
Female	100 (47)	82 (48)	18 (44)	
**Methylated MGMT promoter**				0.85[c]
Positive	113 (53)	90 (53)	23 (56)	
Negative	99 (47)	81 (47)	18 (44)	
**IDH1/2 mutation**				0.57[c]
Positive	17 (8)	14 (8)	3 (7)	
Negative	192 (91)	155 (91)	37 (90)	
Not available	3 (1)	2 (1)	1 (3)	

MGMT, O^6-methylguanine-DNA methyltransferase; IDH, isocitrate dehydrogenase.
Unless otherwise indicated, data represent the number of patients (percentages).
[a] Data are means ± SD.
[b] Calculated with the independent samples t-test.
[c] Calculated with Fisher's exact test.

Supplementary Table 2. MRI scan parameters.

	**Magnetom Verio**	**Magnetom Skyra**	**Ingenia CX 3.0T**	**Discovery MR 750w**
Field strength (T)	3.0	3.0	3.0	3.0
Head coil channel	32	64	32	32
**T1WI**				
TR (ms)	1670	1670	8.6	8.6
TE (ms)	2.8	2.8	4.6	3.2
FA (°)	9	9	8	12
NEX	1	1	1	1
Matrix	256×232	256×232	252×250	256×256
Section thickness (mm)	1	1	1	1
Intersection gap (mm)	0	0	0	0
FOV (mm)	227×250	227×250	250×250	256×256
**T2 FLAIR**				
TR (ms)	8000	8000	9000	9000
TE (ms)	105	105	110	92.3
TI (ms)	2800	2500	2500	2446
FA (°)	150	150	90	160
NEX	1	1	1	1
Matrix	384×244	384×244	352×186	320×224
FOV (mm)	199×220	199×220	220×220	220×220
Section thickness (mm)	4	4	5	5
Intersection gap (mm)	0.8	0.8	1.0	1.0
**DSC-PWI**				
TR (ms)	1600	1600	1483.2	1500
TE (ms)	30	30	30	29.2
FA (°)	90	90	40	60
NEX	1	1	1	1
Matrix	128×128	128×128	128×126	100×100

FOV (mm)	240×240	240×240	224×224	240×240
Section thickness (mm)	6.0	6.0	5.0	5.0
Voxel size (mm^3)	1.88×1.88×6.00	1.88×1.88×6.00	1.88×1.85×6.00	2.40×2.40×5.00
Total acquisition time (sec)	1 min 43 sec	1 min 43 sec	1 min 41 sec	1 min 36 sec

MRI, magnetic resonance imaging; T1WI, T1-weighted imaging; FLAIR, fluid-attenuated inversion recovery; TR, repetition time; TE, echo time; FA, flip angle; NEX, number of excitations; FOV, field of view; DCE MRI, dynamic contrast-enhanced MR imaging.

Supplementary Table 3. Parameters for the model training.

Parameter name	Value
Loss function	Dice and cross-entropy
Optimizer	Adam with learning rate weight decay
Initial Learning rate	0.0003
Weight initialization	Kaiming He
Data augmentation	Rotation, gamma, scaling, elastic deform, mirror
Batch size	2
Patch size	128×128×112

Supplementary Figure

Supplementary Figure 1. Training curves for the deep learning models. With increasing iterations, the training models' loss function steadily decreased while Dice score (moving averaged) steadily increased for both the conventional MRI model (A) and multiparametric MRI (B) model. Training terminated automatically after reaching the plateau at which the change in loss function (Dice loss + cross entropy) was minimal.

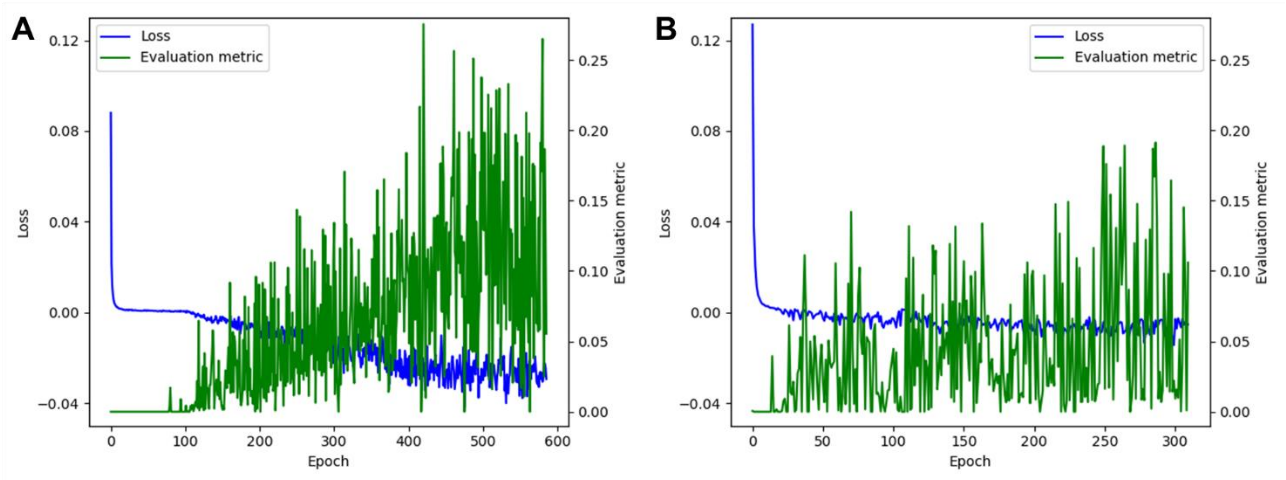

Supplementary Figure 2. Receiver operating characteristic curves showing sensitivities and specificities at various probability cutoff values in the test set. The probability cutoff was decreased from 1 (index 1) to 0 (index 30) by 1/30 (Note: Some probability cutoffs resulted in the same true positive and false positive rates). At the probability cutoff of 0.5 (red box), the sensitivity was doubled from 40% to 80% with the addition of nCBV, while the specificity was compromised nonsignificantly. AUC values of the conventional and multiparametric MRI models were 0.47 and 0.52, respectively.

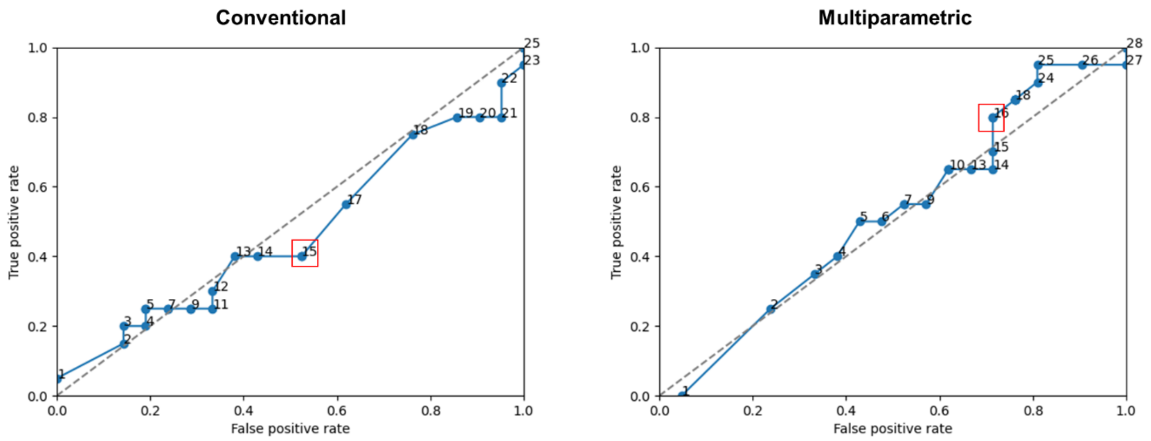

Supplement: Supplementary file 1 — Supplementary Information. [file 41598_2023_41171_MOESM1_ESM.pdf]
